# A Smartphone-Based Intervention for Anxiety and Depression in Racially and Ethnically Diverse Adults (EASE): Protocol for a Randomized Controlled Trial

**DOI:** 10.2196/40713

**Published:** 2022-12-05

**Authors:** Lorra Garey, Michael J Zvolensky, Matthew W Gallagher, Anka Vujanovic, Darla E Kendzor, Lancer Stephens, Marshall K Cheney, Ashley B Cole, Krista Kezbers, Cameron T Matoska, Jillian Robison, Audrey Montgomery, Christopher V Zappi, Michael S Businelle

**Affiliations:** 1 HEALTH Institute University of Houston Houston, TX United States; 2 Department of Psychology University of Houston Houston, TX United States; 3 Department of Behavioral Science MD Anderson Cancer Center University of Texas Houston, TX United States; 4 Texas Institute for Measurement, Evaluation, and Statistics University of Houston Houston, TX United States; 5 TSET Health Promotion Research Center Stephenson Cancer Center Oklahoma City, OK United States; 6 Department of Family and Preventive Medicine Health Sciences Center University of Oklahoma Oklahoma City, OK United States; 7 College of Public Health Health Sciences Center University of Oklahoma Oklahoma City, OK United States; 8 Oklahoma Shared Clinical and Translational Research Resources Health Sciences Center University of Oklahoma Oklahoma City, OK United States; 9 Department of Health and Exercise Science University of Oklahoma Norman, OK United States; 10 Department of Psychology Oklahoma State University Stillwater, OK United States

**Keywords:** COVID-19, just-in-time adaptive intervention, anxiety, depression, mHealth, minority populations, death, behavioral, care, mobile application, app, public health, symptoms, risk

## Abstract

**Background:**

Clear health disparities have emerged in the rates of COVID-19 exposure, hospitalization, and death among Black, Hispanic, and American Indian (BHAI) individuals, relative to non-Hispanic White (NHW) individuals. BHAI populations have been disproportionately affected by lower behavioral health access and heightened negative mental health outcomes during the pandemic.

**Objective:**

This project directly addresses health disparities in access to behavioral health care during the COVID-19 pandemic among BHAI populations via an adaptation of the established, initially validated, low-cost, mobile app Easing Anxiety Sensitivity for Everyone (EASE) among individuals with symptoms of elevated anxiety or depression or both.

**Methods:**

The EASE trial is a 2-arm, prospective, randomized, blinded-assessor study with intention-to-treat analysis. Participants (N=800; n=200, 25%, Black; n=200, 25%, Hispanic; n=200, 25%, American Indian; and n=200, 25%, NHW) are randomized to receive either EASE or an active comparison condition for anxiety and depression. Participants compete an online prescreener, an enrollment call to provide informed consent, a baseline survey, a 6-month intervention period, and 3- and 6-month postbaseline assessments. Select participants also complete a 3- and 6-month postbaseline qualitative interview via phone or an online platform (eg, Zoom). Participants complete 2 scheduled daily ecological momentary assessments (EMAs) during the 6-month study period. These twice-daily EMAs guide a just-in-time approach to immediate, personalized behavioral health care.

**Results:**

Outcomes include reductions in anxiety and depressive symptoms and functional impairment at 3 and 6 months postrandomization. We also will examine putative mechanisms (eg, anxiety sensitivity [AS] and COVID-19–specific stress and fear) of the intervention effects. Further, as treatment effects may differ across sociocultural factors, perceived discrimination, social support, and socioeconomic status (SES) will be evaluated as potential moderators of treatment effects on the primary outcomes. Process evaluation using data collected during the study, as well as individual interviews with participants, will complement quantitative data.

**Conclusions:**

Data from this efficacy trial will determine whether EASE successfully improves symptoms of anxiety and depression and whether these improvements outperform an active comparison control app. If successful, findings from this study have the potential to decrease anxiety and depression symptoms among vulnerable populations determined to be most at risk of exacerbated, long-lasting negative health sequelae. Data from this study may be used to support an implementation and dissemination trial of EASE within real-world behavioral health and social service settings.

**Trial Registration:**

ClinicalTrials.gov NCT05074693; https://clinicaltrials.gov/ct2/show/NCT05074693

**International Registered Report Identifier (IRRID):**

DERR1-10.2196/40713

## Introduction

Significant racial/ethnic health disparities have been identified in the United States [1] related to COVID-19 [2]. Indeed, the rates of COVID-19 infection, hospitalizations, and death are disproportionately higher among Black, Hispanic, and American Indian (BHAI) individuals relative to non-Hispanic White (NHW) persons [1,3-6]. BHAI individuals are also more likely to experience COVID-19–related stress relative to NHW persons [7]. Recent data from April 27 to May 9, 2022, indicate that 31.2% of Black and 34.7% of Hispanic individuals report current symptoms of an anxiety or depressive disorder [8]. These estimates are in stark contrast to the estimated 11.3% of Black and 10.3% of Hispanic individuals who reported symptoms of an anxiety or depressive disorder prior to the COVID-19 pandemic from January to July 2019 [8]. Although NHW individuals experience comparable, albeit slightly lower, rates of anxiety or depression (30.0% from April 27 to May 9, 2022) [8], the racial/ethnic disparity gap for mental health outcomes is widening. To illustrate, data from January to July 2019 found a 0.2%-1.2% difference in elevated anxiety or depression symptoms between NHW and Black and Hispanic persons, with NHW individuals evincing higher prevalence rates of these symptoms (11.5%) [8]. The inverse pattern is now prevalent, with Black and Hispanic persons reporting higher rates of these symptoms by 1.2%-4.7% [8,9]. Although postpandemic onset mental health data for American Indians in the United States is lacking, American Indian community leaders have expressed continued concern for the health of their constituents, given their propensity for worse physical and mental health outcomes related to COVID-19 [10,11]. Pre-existing inequities related to social determinants of health (eg, lower access to mental health care and therefore less treatment engagement [12,13]) and the effects of racism [14] have likely contributed to and exacerbated these disparities and may compound the severity of emerging COVID-19–related mental health disparities [7]. Emerging COVID-19–related disparities in mental health symptoms are likely to widen without proper early intervention tactics.

The ongoing COVID-19 pandemic has led to increases in stress, including stress related to unemployment, homelessness, food insecurity, and lack of adequate health care resources. The COVID-19 stress burden may have a particularly pernicious influence in persons already struggling with greater stress exposure and pre-existing psychopathology [15]. Theoretically, higher degrees of COVID-19 stress may influence patterns of social, emotional, and neurobiological development, facilitating the rapid detection of potential threats, and, by extension, increase the risk for multiple forms of psychopathology. A transdiagnostic approach to intervention development and deployment could serve as an effective treatment method. This approach relies on identifying the fundamental processes underlying multiple psychopathologies [16] and may be particularly effective and efficient at mitigating emerging mental health disparities arising from the pandemic.

Anxiety sensitivity (AS), defined as the fear of anxiety symptoms, including bodily sensations [17], has been identified as a key vulnerability factor for anxiety [17-20], depression [21-23], substance use [24-26], and other psychopathology symptoms [27-29]. Theoretical models posit that individuals with high AS are more likely to attend to bodily sensations that are associated with anxiety, such as respiratory symptoms, stomach distress, fatigue, and body aches, and to misinterpret these symptoms as dangerous or catastrophic [30,31]. These interpretations of bodily sensations can lead to increased anxiety and perpetuate a cycle of increased attention to and misinterpretation of bodily cues [31-33]. This process may eventually lead to avoidance and increased symptoms of anxiety, stress, and depression, with potential to exacerbate stress on the body systems, further compromising the immune system and placing certain individuals at greater risk for more severe psychopathology [34]. This maladaptive cycle may be particularly relevant to BHAI populations during and following the pandemic, given their increased likelihood of COVID-19 exposure [5,35-39], challenges with enacting behaviors to reduce the likelihood of infection, concerns about the increased likelihood of worse outcomes if infected, and greater pandemic-related stressors (eg, job loss [40], income reduction [41-43], childcare needs [44,45], and discrimination). Further, because it has been shown previously that some minority groups experience higher levels of distress expressed somatically [46], AS is particularly relevant because it could amplify the threat response to somatic perturbation.

Importantly, AS is malleable in response to psychosocial interventions [47], making it a prime mechanism to target in prevention/intervention programs. Reductions in AS improve clinical outcomes among clinical and nonclinical populations [47], reductions in AS have been shown to be associated with positive outcomes in smokers who are motivated to quit [48], and AS can be effectively engaged through in-person and digitally delivered methods [49,50]. This is a critical consideration, given the overwhelming stress that the pandemic has placed on the health care system and the current paradigm shift toward providing care remotely [51,52]. In the context of mental health, digital health (eg, mobile health [mHealth]), telemedicine/telehealth, and health IT (eg, mobile phones, wearable sensors) can be used to develop scalable interventions that offer mental health care that is personalized to meet the unique needs of patients [53-55] and thus reduce the burden on the health care system. Indeed, mHealth interventions have strong potential for a broad reach, high accessibility, and widespread dissemination and serve as a potential vehicle to provide access to evidence-based mental health treatments.

A recent meta-analytic review found that mHealth interventions for anxiety and depression reduction may lead to significant within-person reductions in anxiety and depression, and the reduction in anxiety was greater than what was reported by control participants [56]. The majority of the reviewed mHealth apps (67%), however, focused on strict cognitive behavioral therapy (CBT) and did not consider the potential of using a transdiagnostic approach to negative affective symptom reduction. Using an mHealth framework to deliver mHealth AS reduction treatment could address both individual- and system-level barriers to accessing effective mental health treatment during and after the pandemic and result in reduced anxiety and depressive symptoms by effectively engaging an underlying mechanism implicated in both conditions.

This study capitalizes on our expertise in mHealth interventions, mental health treatments, and health disparities research. We propose to test an accessible mHealth AS reduction treatment that incorporates a just-in-time treatment approach to address the mental health sequelae resulting from the COVID-19 pandemic. The primary aim of this study is to test the effects of a novel, smartphone-delivered intervention (Easing Anxiety Sensitivity for Everyone [EASE]) aimed at reducing mental health disparities across racial/ethnic minority groups that have been exacerbated by the COVID-19 pandemic in a large, racially/ethnically diverse sample of adults with clinically significant anxiety or depression. The comparison condition is a time-matched real-world treatment (mindfulness/relaxation-based therapy). Our primary outcomes are reductions in anxiety and depression symptoms and functional impairment at 3 and 6 months postrandomization. Recognizing the clinical importance of mechanistic research to optimize interventions [40], we will also examine putative mechanisms (eg, AS and COVID-19–specific stress and fear) of the intervention effects. Moreover, as treatment effects may differ across sociocultural factors, perceived discrimination, social support, and socioeconomic status (SES; indexed by monthly income) will be evaluated as potential moderators of treatment effects on the primary outcomes.

## Methods

### Ethical Considerations

All participants provide written informed consent electronically after reviewing consent documents with research staff. To protect participant privacy and confidentiality, phone-based appointments are completed by research staff in a secure office at 1 of the 2 collaborating sites. Additionally, participants are assigned an ID number that is used to identify their data throughout the study. Only trained research staff have access to the key that can match participant data to the participant’s name. The key is password-protected on a secure server housed at the collaborating institutions. Participants are compensated up to US $410 for participating in the study and receive compensation via a GreenPhire [57] Mastercard gift card that is loaded by research staff. The Institutional Review Board (IRB) at the University of Houston approved the study (IRB approval no. STUDY00002802) and serves as the Relying IRB for all sites under the National Institutes of Health (NIH) Single IRB policy, and a Data Safety and Monitoring Board provides ongoing oversight.

### Study Design

**Figure 1:**
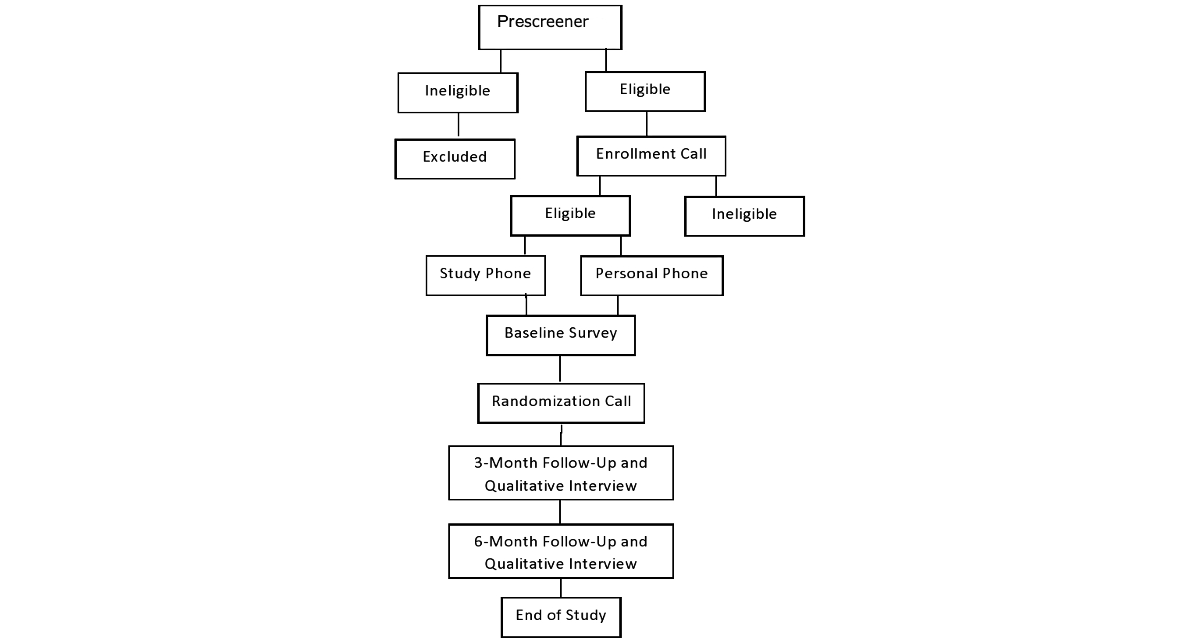
Participant flow diagram.

Adults who report clinically significant anxiety or depression (N=800; racial breakdown: n=200, 25%, Black/African American; n=200, 25%, Hispanic; n=200, 25%, American Indian; and n=200, 25%, NHW individuals) are being recruited and enrolled to participate in a trial on the effects of a novel, smartphone-delivered intervention to address negative mood symptoms and reduce COVID-19–related mental health disparities. Participants are recruited via community organizations and social media and internet outlets (eg, Craigslist, Facebook). Interested individuals complete an online prescreener assessment, where they are asked to answer basic demographic questions, questions about their phone use, and levels of anxiety and depression they are experiencing via the Overall Anxiety Severity and Impairment Scale (OASIS) [58] and the Overall Depression Severity and Impairment Scale (ODSIS) [59]. Those eligible at the prescreener complete an enrollment call to assess for full eligibility and receive instructions on how to download the app to their smartphone to complete the baseline assessment. Participants who do not own a smartphone that is compatible with the Insight app [60] are mailed a study phone and contacted by study staff once the study phone arrives. Following completion of the baseline assessment, research staff contact participants to schedule their randomization phone call. During the randomization call, research staff randomly assign the participant to either the (1) EASE or the (2) mindfulness/relaxation-based comparison condition. Participants then engage with the assigned intervention for 6 months following randomization. Participants complete follow-up assessments at 3 and 6 months postrandomization, which includes self-report measures, and a randomly selected subset of the sample completes a qualitative interview focused on app use and experiences with the app. Additionally, participants are prompted to complete ecological momentary assessments (EMAs) twice daily throughout the 6-month postrandomization period. See Figure 1 for the participant flow diagram.

### Specific Aims and Hypotheses

This study has 2 specific aims:

- To use a randomized controlled trial (RCT) design to compare the effects of EASE to a mindfulness/relaxation-based comparison group on anxiety and depression symptoms, and functional impairment at 3-month and 6-month follow-up. We hypothesize that those assigned to EASE will show greater reductions from baseline to follow-ups in OASIS [58] and ODSIS [59] scores and also greater reductions in functional impairment in daily responsibilities (eg, work performance, household maintenance, social interactions, and relationships) [61] relative to the comparison group (hypothesis 1 [H1]). We also hypothesize that the effectiveness of EASE (as assessed by slope changes from baseline to follow-ups in outcomes listed in H1) will be similar across racial/ethnic groups (H2).
- To examine the mechanisms of action at 3- and 6-month follow-ups (H3) and moderators of treatment outcomes at 3- and 6-month follow-ups (H4). In testing the putative mechanisms of action, we hypothesize that the intervention effects on study outcomes will be mediated by reductions in AS and changes in COVID-19–related stress and fear. To determine possible moderators, we will test whether treatment effects vary as a function of perceived discrimination (worse intervention outcomes), social support (better intervention outcomes), or SES (lower SES associated with worse outcomes).

This study has 2 exploratory aims:

- To identify opportunities to improve the efficacy, reach, and adoption of EASE through qualitative interviews
- To use daily EMAs to obtain a granular understanding of the course (eg, changes in anxiety/depression symptoms and fear of COVID-19) and sequelae (eg, employment status, daily activities, substance use) of the COVID-19 pandemic and health behaviors affected by COVID-19 (ie, physical activity, pain experience, sleep) among those who do and do not contract the virus

### Participants

Participants will be 800 adults (n=200, 25%, Black/African American; n=200, 25%, Hispanic; n=200, 25%, American Indian; and n=200, 25%, NHW individuals) who report current clinically significant anxiety or depression. All participants must meet the following eligibility criteria: (1) have clinically significant anxiety or depression symptoms, as evinced by a score of 8 or higher on OASIS [58] or a score of 8 or higher on ODSIS [59]; (2) be aged 18 years or older; (3) self-identify as Black, Hispanic, American Indian, or NHW (singular or multiracial identification); (4) be willing to complete all study surveys/assessments; and (5) score 4 or more on the Rapid Estimate of Adult Literacy in Medicine-Short Form (REALM-SF) indicating an English literacy level higher than the sixth grade. Exclusion criteria include the following: (1) identifying with a race/ethnicity for which the corresponding study cell has been filled, (2) significant cognitive impairment evinced by a score of 8 or higher on the 6-Item Cognitive Impairment Test (6CIT) [62], and (3) inability to read or understand English at the sixth-grade or higher level.

### Procedures

Enrollment to the RCT began in December 2021 and, as of this writing, is in the recruitment phase. Enrollment, randomization, intervention delivery, and assessments (EMAs and follow-ups) are completed remotely via a smartphone or phone call with trained research staff. Both study conditions receive the same set of questionnaires at each assessment (see Tables 1 and 2 for a full list and timeline of study measures). These questionnaires assess anxiety or depression symptoms, functional impairment, general and COVID-19–specific affect constructs, and sociocultural factors.

Interested individuals complete an initial 5-minute online screener via Research Electronic Data Capture (REDCap; Vanderbilt University) to assess age, racial/ethnic identity, clinical anxiety and depression, state of residence, and willingness to complete study assessments. Those deemed eligible at the online screener complete an enrollment call with research staff that lasts approximately 30 minutes. During the enrollment call, participants provide informed consent and are informed of the purpose, goals, and procedures of the study. Participants are also further assessed for eligibility (ie, English literacy and fluency, and cognitive impairment). Those found eligible are instructed on how to download the smartphone-based app that will administer assessment and intervention content (ie, Insight app) [60] during the enrollment call. Eligible participants who do not have a smartphone that is compatible with the study app are mailed a study smartphone to use during study participation and are contacted by study staff once the phone is received. After participants download the study app to their personal or study-provided phone, they are oriented on how to use the app to complete the baseline assessment and instructed to complete it within the next 7 days. The smartphone-based baseline assessment takes approximately 20 minutes to complete. Participants who have not completed the baseline assessment within 48 hours are contacted by study staff via text, phone, or email to remind them to complete the assessment.

**Table 1:** List and timeline of study measures.

Measure	Screener	Enrollment	Baseline	Follow-ups
Demographic Questionnaire	X^a^	N/A^b^	X	N/A
6CIT^c^ [62]	N/A	X	N/A	N/A
REALM-SF^d^ [63]	N/A	X	N/A	N/A
Demographic/Background Information Questionnaire	X	N/A	N/A	N/A
Functional Impairment Related to Anxiety and Depression	N/A	N/A	X	X
EDS^e^ [64]	N/A	N/A	X	X
CRBS^f^ [65]	N/A	N/A	X	X
F-SozU K-6^g^ [66]	N/A	N/A	X	X
OASIS^h^ [58]	X	N/A	X	X
ODSIS^i^ [59]	X	N/A	X	X
SSASI^j^ [67]	N/A	N/A	X	X
Fear of COVID-19 [68]	N/A	N/A	X	X
COVID-19 Psychological Impact Survey	N/A	N/A	X	X

^a^X: applicable.

^b^N/A: not applicable.

^c^6CIT: 6-Item Cognitive Impairment Test.

^d^REALM-SF: Rapid Estimate of Adult Literacy in Medicine-Short Form.

^e^EDS: Everyday Discrimination Scale.

^f^CRBS: Coronavirus Racial Bias Scale.

^g^F-SozU K-6: 6-Item Perceived Social Support Questionnaire.

^h^OASIS: Overall Anxiety Severity and Impairment Scale.

^i^ODSIS: Overall Depression Severity and Impairment Scale.

^j^SSASI: Short-Scale Anxiety Sensitivity Index.

**Table 2:** List and timeline of EMAs^a^.

Assessment			Daily diary		Sunday morning diary		Report distress		COVID-19 symptoms
Sleep			X^b^		N/A^c^		N/A		N/A
Alcohol consumption			N/A		N/A		N/A		N/A
Cigarette consumption			X		N/A		N/A		N/A
Marijuana use			X		N/A		N/A		N/A
Fast-food consumption			X		N/A		N/A		N/A
Other substance use			X		N/A		N/A		N/A
SSASI^d^			X		N/A		N/A		N/A
Prescription medication consumption			X		N/A		N/A		N/A
**Discrimination experience**									
	Affect	X		N/A		X		N/A	
	Pain level	X		N/A		X		N/A	
	Ability to cope with stress	X		N/A		X		N/A	
	Social support	X		N/A		N/A		N/A	
	Physical activity	X		N/A		N/A		N/A	
	Location	N/A		N/A		X		N/A	
	Self-rated health questionnaire	N/A		X		N/A		N/A	
	OASIS^e^	N/A		X		N/A		N/A	
	SSASI	N/A		X		N/A		N/A	
	Current COVID-19 symptoms	N/A		N/A		N/A		X	
	Exposure to someone with COVID-19	N/A		N/A		N/A		X	

^a^EMA: ecological momentary assessment.

^b^X: applicable.

^c^N/A: not applicable.

^d^SSASI: Short-Scale Anxiety Sensitivity Index.

^e^OASIS: Overall Anxiety Severity and Impairment Scale.

Research staff are automatically notified via an encrypted email once a participant completes the baseline assessment. Once notified, research staff call the participant and complete the randomization process with the participant. During the randomization call, participants are randomly assigned to an intervention condition: (1) EASE or (2) mindfulness-based comparison; see Table 3 for a comparison of treatment components. Specifically, participants are randomized using permuted block randomization to allocate them to a treatment condition within each racial/ethnic category (EASE=100, 12.5%, per racial/ethnic group; mindfulness/relaxation-based comparison=100, 12.5%, per racial/ethnic group). The study statistician (author MWG) set up the randomization schema tables (stratified by racial/ethnic identity and sex assigned at birth) in REDCap and has no contact with participants, thereby preventing experimenter bias in randomization. Only the study project manager can see and access the tables once uploaded to REDCap; all other study team members use the REDCap randomization module, which hides the allocation until randomization is prompted.

**Figure 2:**
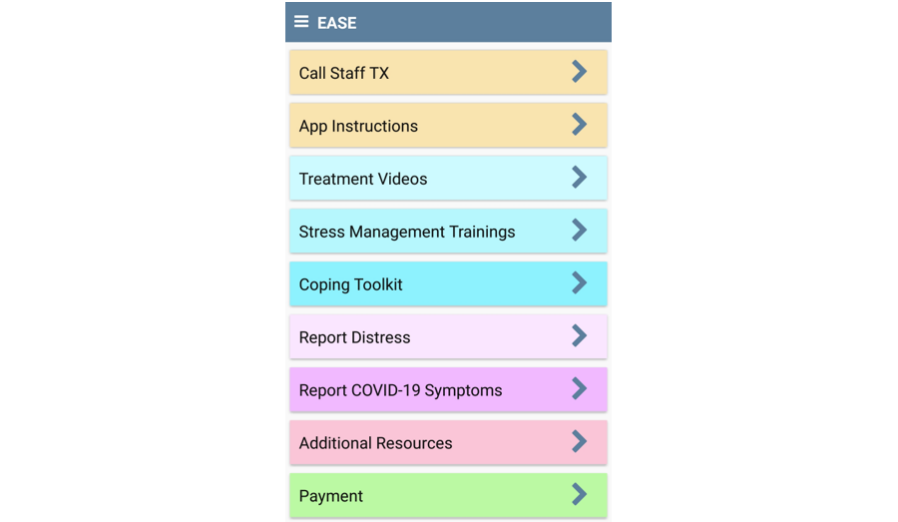
EASE app home screen. EASE: Easing Anxiety Sensitivity for Everyone; TX: Texas.

Following randomization, research staff provide participants with a code that enables them to access condition-specific intervention materials. Next, trained research staff provide a brief app orientation to the participant on how to use the app and its components, and answer any questions the participant may have. Research staff also provide a brief orientation on how to report distress and COVID-19 symptoms via the app (both groups). The day after the randomization call, all participants begin to receive 2 prompts to complete daily EMAs, one 30 minutes after waking and one 75 minutes before bedtime. Daily EMAs take approximately 2 minutes to complete. Research staff monitor participant EMA completion rates weekly. When a participant’s completion rate significantly decreases, a staff member contacts the participant via text, phone, or email to remind them of the importance of completing the daily EMAs. Participants are informed that they can contact study staff at any point during the trial by pressing the “Call Staff TX” button at the top of the app home screen if they experience technical difficulties (see Figure 2). Participants are encouraged to use the app daily for 6 months, particularly when they are experiencing distress.

Participants complete follow-up smartphone-based, self-report assessments at 3 and 6 months postrandomization. Additionally, research staff contact participants selected through a modified quota sampling strategy [69] at 3 and 6 months postrandomization to complete a brief qualitative interview to assess their experience with the app and to solicit suggestions on ways to improve the app content, delivery, or reach potential. The qualitative interview uses a semistructured interview guide and is audio-recorded for transcription. The 3- and 6-month self-report assessments each require approximately 20 minutes to complete, and the 3- and 6-month qualitative interviews take approximately 30 minutes to complete. Data gathered through the qualitative interviews will be used to identify opportunities to improve the efficacy, reach, and adoption of the EASE app.

Regarding compensation, all participants who enroll in the study receive a GreenPhire [57] Mastercard gift card to facilitate paying them for completing study surveys. GreenPhire offers an auditable mechanism for all study payments. Participants receive US $20 for completing the baseline assessment and US $50 for completing each of the 3- and 6-month follow-ups. Additionally, participants are compensated for completion of EMAs (2 per day × 30 days = 60 monthly EMAs). Specifically, those who complete 50%-74% of prompted EMAs each month receive US $20, those who complete 75%-89% of assessments receive US $30, and those who complete 90% or more of their EMAs receive a total of US $40 for that month. Participants can click the “Payment” button on the app home screen at any time for an up-to-the-moment summary of EMAs presented and completion rate (see Figure 2). Payments for completing EMAs are loaded onto GreenPhire cards each month. Participants are not compensated for accessing on-demand app features or completing treatment components. Following completion of study participation, participants who borrowed a study smartphone are mailed a prepaid envelope and instructed to return the phone.

**Table 3:** Comparison of treatment components.

App components			EASE^a^	Control
EMA^b^			X^c^	X
COVID-19 monitoring and intervention			X	X
Distress reporting			X	X
App instructions			X	X
AS^d^ psychoeducation			X	N/A^e^
Interoceptive exposure exercises			X	N/A
**On-demand tips and exercises**				
	General relaxation tips	X		X
	Coping with mood	X		N/A
	Coping with stress and anxiety	X		N/A
	Coping with COVID-19	X		N/A
	Coping with loss and grief	X		N/A
	Coping with discrimination	X		N/A
	Videos for distraction	X		N/A
	Interoceptive exposure	X		X
	Guided relaxation exercises	X		N/A
	Inspirational messages	N/A		
	Challenge unhelpful thoughts	N/A		
Tailored treatment messages in real time			X	N/A
State and national resources for needs			X	N/A
Compensation earned			X	X

^a^EASE: Easing Anxiety Sensitivity for Everyone.

^b^EMA: ecological momentary assessment.

^c^X: applicable.

^d^AS: anxiety sensitivity.

^e^N/A: not applicable.

Event-sampling EMAs are self-initiated by the participant. Specifically, all participants are instructed to self-initiate an event-sampling EMA to record distress and new COVID-19 symptoms. Participants are instructed to answer questions based on immediate thoughts/feelings when they initiate an event-sampling EMA. EASE participants receive unique intervention content when they report elevated depression or anxiety symptoms. Further, our previously developed COVID-19 risk assessment and detection algorithm [70] automatically advises participants in both conditions to get a test for COVID-19 when elevated risk is detected. In addition, the app immediately triggers an automated and encrypted email to study staff to call the participant and assist with obtaining a COVID-19 test (if desired by the participant). All EMAs are date-, time-, and location-stamped for future analyses.

### Study Conditions

#### Easing Anxiety Sensitivity for Everyone

EASE integrates standard CBT with AS reduction. EASE provides (1) standard treatment on a schedule, (2) scheduled and cued interoceptive exposure sessions, (3) on-demand intervention content (ie, ways to cope with symptoms of anxiety and depression, cognitive restructuring exercises, guided relaxation videos), (4) tailored treatment messages based on responses to EMA items, and (5) additional resources. All interactions with the app are date-, time-, and location-stamped for future analyses.

##### Treatment on a Schedule

EASE includes 18 four- to six-minute intervention videos (adapted from our previous interventions [71-75] and recorded by racially/ethnically diverse actors) that are delivered via a smartphone over the first 3 months of the intervention period. Specifically, new videos become available during the morning daily EMAs on Mondays and Thursdays. Participants have the option to watch the videos immediately or later by clicking the “Treatment Videos” button (see Figure 2). Videos can be watched as many times as desired, and the app records the date/time/location when each video is watched (both initiation and completion).

The videos cover topics including the role of AS in interoceptive stress, the CBT model of anxiety/depression and interoceptive stress, procedures for each intervention module, coping mechanisms for negative emotions (eg, chronic stress due to sociocultural stressors), fear avoidance hierarchy for specific stressors (eg, bodily stress or stress burden due to experiences of racism or discrimination), interoceptive exposure techniques, the relationship between COVID-19 and anxiety/depression, and use of AS reduction strategies to mitigate the impact of COVID-19–related stress on mental health. Videos also present known disparities regarding COVID-19 and the potential impact on mental health disparities. Finally, several videos focus on relaxation training to help mitigate the impact of stress related to discrimination, general life events, and COVID-19–specific stressors on heightened symptoms of anxiety and depression. See Table 4 for a list of video titles.

**Table 4:** List of video titles.

EASE^a^ group			Active comparison group	
Video number	Title	Video number		Title
1	Introduction to the Program	1		What Is Mindfulness
2	CBT^b^ Model & Example	2		Mindfulness vs Meditation
3a	General Stressors	3		Levels of Mindfulness
3b	Race/Discrimination Stressors	4		The WHAT Skills
4	AS^c^ & Interoceptive Stress	5		The HOW Skills
5	Interoceptive Exercises	6		Living Mindfully
6	Present-Focused Exercise	7		Mindfulness and Acceptance
7	Interoceptive Exercises (CON’TD)	8		Diaphragmatic Breathing Exercise
8	Unhelpful Thinking	9		Body Scan Exercise
9	Thinking Flexibly	10		Walking Mindfully Exercise
10	Avoidance & Exposure	11		Eating Mindfully Exercise
11	Opposite Action	12		Sleep and Mindfulness Exercise
12	Diaphragmatic Breathing	13		Science of Mindfulness
13	Values	14		5-4-3-2-1
14	Grief/Loss and Control	15		STOP Practice
15	Grounding	16		Leaves on a Stream
16	Self-Care & Compassion	N/A^d^		N/A
17	Review Video	N/A		N/A
18	Next Steps	N/A		N/A

^a^EASE: Easing Anxiety Sensitivity for Everyone.

^b^CBT: cognitive behavioral therapy.

^c^AS: anxiety sensitivity.

^d^N/A: not applicable.

##### Exposure Exercises

To target AS, graduated exposure to anxiety and distress-provoking situations and response prevention is introduced, reviewed, and practiced via EASE videos and the “Stress Management Trainings” button. Participants learn to manage negative affect, stress, and uncomfortable physiological symptoms during exposure exercises without acting on acute motivation to suppress distress. These exposure exercises were created and pilot-tested as part of our previous work on smartphone-delivered AS reduction [72]. The exercises include overbreathing, straw breathing, running in place, chair spinning, and head rush. EASE participants are reminded to practice the interoceptive exercises during treatment videos and are asked whether they would like to practice stress management training 2 times per day (during morning and evening EMAs). Participants can access the exposures by clicking the “Stress Management Trainings” button on the app home screen (see Figure 2). When this button is clicked, the app explains the purpose of the exercise and assesses the current level of distress (0-100 scale) and physical sensations (1-7 scale). Next, the app randomly selects 1 of the 5 exercises and explains how to complete it. A 1-minute countdown timer is initiated, and the participant completes the exercise. Following the exercise, the app reassesses the level of distress and physical sensations. The app suggests repeating the exercise up to 3 additional times if the current reported distress is greater than 50 on a 1-100 scale. This strategy aims to increase habituation to feared sensations. Participants are encouraged to continue practicing these exposures daily.

##### On-Demand Features

**Figure 3:**
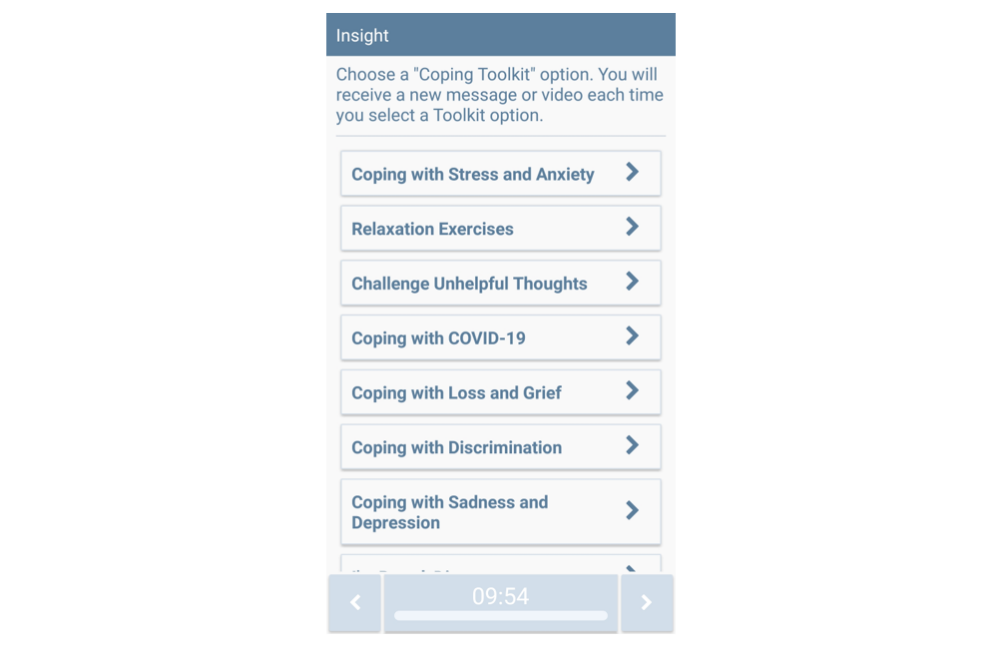
"Coping Toolkit" menu of on-demand intervention content.

Participants have access to a “Coping Toolkit” menu of on-demand intervention content (see Figure 3). Specifically, participants can access (1) suggestions on ways to cope with stress and anxiety, (2) guided relaxation exercise videos, (3) exercises focused on challenging automatic thoughts, (4) suggestions for coping with COVID-19–related stress, (5) coping with loss and grief, (6) coping with discrimination, and (7) coping with sadness and depression. In addition, participants are instructed to click the “I’m Bored, Distract Me” button to gain access to brief funny or cute videos. Further, participants are prompted to press the “Report Distress” button when they experience elevated stress, anxiety, or depression (see Figure 2). The Report Distress assessment includes questions about mood and feelings, and the app provides tailored content based on current anxiety, depression, and self-reported ability to cope with current emotions. The “Report COVID-19 Symptoms” button assesses symptoms that are consistent with COVID-19. When symptom clusters that are consistent with COVID-19 are reported, participants are informed that they should get tested and asked whether they would like help with finding a testing center. An encrypted email is sent to study staff in real time when participants report symptoms consistent with COVID-19. The “Additional Resources” button contains links to state and federal resources focused on COVID-19, housing, food, and job placement.

##### EMAs With Tailored Real-Time Treatment Messages

During the 6-month intervention period, a tailored message is delivered at the end of each scheduled EMA (2 per day). Specifically, when participants provide a pattern of responses that are suggestive of heightened emotional distress (eg, current depression or anxiety/stress score>5 on a 1-10 scale), they receive a coping message that attempts to address current symptoms (eg, “If you are feeling stressed, take a moment to breathe. Breathe in for 5 seconds, hold it, and breathe out. Stress and bad moods are temporary.” or “Research shows that sunlight can help reduce feelings of depression. Take some time for a walk today.”).

#### Mindfulness-Based/Relaxation-Based Comparison

Participants randomized to the mindfulness/relaxation-based comparison condition have access to some app components that are similar to EASE. The comparison intervention emphasizes the importance of mindfulness in the larger context of mental and physical health and specific skills to improve mindfulness and promote relaxation/stress reduction [76-78]. The 16 treatment videos selected for this treatment were chosen based on their relevance to the target population and potential to offer a real-world comparison. Topics covered in the videos include an introduction to the concept of mindfulness, the difference and similarities between mindfulness and meditation, guided meditations, and guided mindfulness-based exercises (ie, 5-4-3-2-1 exercise, leaves on a stream thought, diaphragmatic breathing). Similar to EASE, during the Monday and Thursday morning EMAs, comparison group participants are asked whether they would like to watch 1 new 5-10-minute mindfulness-based psychoeducation and skill-building videos. Videos also become available weekly via the “Treatment Videos” button. The comparison group does not have access to the stress management training exercises, and EMAs are not paired with tailored real-time treatment messages. The “Report COVID-19 Symptoms” button is fully functional and identical to the EASE version of this feature. The “Report Distress” button is available, but tailored messaging is not delivered at the end of the Report Distress assessment.

### Assessments

Prescreen and enrollment call data are collected via REDCap. Baseline, EMA, and follow-up data are collected using smartphones through the Insight mHealth platform app software [60]. Qualitive follow-up data are collected via phone calls. This approach reduces data entry errors and the need to retain paper copies of raw data. Each question appears on the smartphone screen, and the participant responds by touching their answer on the touch screen. Data are collected via self-report, with the exception of the REALM-SF [63] and the 6CIT [62], which are administered via interviews during the enrollment phone call. See Tables 1 and 2 for a schedule of study measures.

#### Demographics and Screener Questions

Participants are asked to provide standard demographic information (ie, name, contact information, age, sex, race/ethnicity, monthly income used as a measure of the SES [79-83], level of education), current state of residence, comfort with the English language, information about the type of phone they have, and whether they would like to download the app to their personal phone or be provided with a phone with the app predownloaded to it.

#### English Literacy

The REALM-SF [63] is an interviewer-administered checklist in which individuals are asked to read and pronounce 9 common medical terms. Individuals who pronounce ≥4 words correctly are considered to be reading at a reading level higher than the sixth grade (an English literacy level higher than the sixth grade is required to complete EMAs). The REALM-SF is administered by trained research staff during the enrollment call.

#### Cognitive Functioning

The 6CIT is a brief cognitive function test that takes less than 5 minutes to complete and is widely used in primary care settings [62]. The scale uses an inverse score, and questions are weighted to produce a total out of 28. Participants with scores in the normal range (ie, 0-7) are eligible for study participation. Individuals with scores of 8 or higher (ie, mild-to-severe impairment) are excluded from participating in this study.

#### Anxiety

OASIS [58] is a 5-item measure that has demonstrated strong psychometric properties to identify those with clinically significant anxiety symptoms [58]. A score of 8 or higher is indicative of clinically significant anxiety. OASIS is administered during the REDCap prescreen (to screen for eligibility), baseline assessment, and 3- and 6-month postrandomization assessments.

#### Depression

ODSIS [59] is a 5-item measure that has demonstrated strong psychometric properties to identify those with clinically significant depression symptoms [59]. A score of 8 or higher is indicative of clinically significant depression. ODSIS is administered during the REDCap prescreen (to screen for eligibility), baseline assessment, and 3- and 6-month postrandomization follow-up assessments.

#### Functional Impairment

The research team developed items to assess functional impairment related to experiencing anxiety and depression. These items assess the extent to which a person’s relationships, work performance, household maintenance, and social interactions have been negatively affected by depression and anxiety. These items are administered at baseline and 3- and 6-month postrandomization follow-up assessments.

#### Anxiety Sensitivity

The Short-Scale Anxiety Sensitivity Index (SSASI) [67] is a 5-item brief measure of AS derived from the Anxiety Sensitivity Index-3 (ASI-3) that has demonstrated excellent psychometric properties. The SSASI is administered at baseline and 3- and 6-month postrandomization follow-up assessments.

#### COVID-19–Related Stress and Fear

The Fear of COVID-19 [68] scale is a 6-item validated measure to assess emotional fear and the somatic expression of fear related to COVID-19. The COVID-19 Psychological Impact Survey is a 4-item self-report measure of the psychological impacts of COVID-19 and associated risk factors [84]. The survey asks (1) the extent to which participants agree with the statement “I worry about the coronavirus all of the time” (1=strongly disagree to 7=strongly agree), (2) how sad participants feel when they think about coronavirus (0=not at all to 100=extremely), (3) how stressed participants feel when they think about coronavirus (0=not at all to 100=extremely), and (4) the degree to which the participant is stressed due to the COVID-19 pandemic (0=no stress to 100=extreme stress). The Fear of COVID-19 and COVID-19 Psychological Impact Survey measures are administered at baseline and 3- and 6-month postrandomization follow-up assessments.

#### Perceived Discrimination

The Everyday Discrimination Scale (EDS) [64] is a 6-item validated measure of discrimination. The Coronavirus Racial Bias Scale (CRBS) [65] is a 9-item Likert-type scale designed to assess disparities in COVID-19, including the likelihood of contracting COVID-19, lack of appropriate health care, and viewing negative social media posts against certain racial groups. Both measures are administered at baseline and 3- and 6-month postrandomization follow-up assessments.

#### Social Support

The 6-Item Perceived Social Support Questionnaire (F-SozU K-6) [66] is a self-report measure of the perceived availability of social support [66]. This measure is administered at baseline and 3- and 6-month postrandomization follow-up assessments.

#### Ecological Momentary Assessments

The EMA methodology used for this study is similar to that used in our previous studies and by other researchers [74,75,85-95]. Each EMA asks about current emotional, physical, and behavioral symptoms and takes approximately 2 minutes to complete. Two types of EMAs are used: time-based sampling (ie, daily diary) and event sampling. Time-based EMAs are prompted and initiated by the smartphone 2 times per day during the 6-month study period (ie, morning daily diary 30 minutes after the participant’s preset wake time, end-of-day daily diary 75 minutes before the participant’s preset bedtime). The phone rings and vibrates to cue these EMAs for 5 minutes (ie, alternating between 30-second alerting and silence periods). If the participant does not respond to the daily diary prompts, the EMA is automatically rescheduled (up to 3 times during morning assessments and twice during evening assessments) 15 minutes later. EMA questions ask about current symptoms of anxiety and depression, medication use, current thoughts/affect/behaviors, social support, discrimination experiences, sleep quality, substance use, fast-food consumption, current location, fear of COVID-19, employment status, daily activities, and current COVID-19 symptoms. Participants are asked to rate their emotional state by indicating the extent to which they agree or disagree with each of 15 statements (using a 5-point scale from strongly disagree to strongly agree): I feel stressed, happy, angry, tired, afraid, relaxed, sad, bored, worried, lonely, calm, depressed, overwhelmed, energetic, and anxious (from the circumplex model of affect) [96]. Participants also indicate their current environment/social setting. In addition, participants are prompted to watch treatment videos twice per week (“A great way to get your day started is to watch a NEW short video that provides information and tips for handling stress. Would you like to watch the brief new video now? Note: Each video provides new information.”) and complete stress management exercises in the app (“Would you like to complete an exercise right now?”).

### Data Analysis

#### Overview

Hypotheses for aims 1 and 2 will be examined using latent growth models (LGMs) [97], multilevel structural equation models (MSEMs) [98], and latent difference score models [99] using MPlus 8.0 [100]. Data will first be examined for multivariate normality and outliers to determine the most appropriate estimator. The maximum likelihood (ML) estimator will be used if data are approximately normal. Robust maximum likelihood (MLR) will be used if the data are not multivariate normal. LGMs will generally be used to model outcomes collected at major assessments. MSEMs will be used to further examine the longitudinal course of outcomes and hypothesized mechanisms of action as an MSEM is ideally suited for disentangling within and between individual variance in order to more precisely estimate main effects, lagged effects, and indirect effects in intensive longitudinal designs. Evaluation of the model fit in the LGMs/MSEMs will be examined using fit diagnostics (ie, standardized residuals) and common fit statistics (ie, root-mean-square error of approximation) following the associated cut-off criteria recommended by Hu and Bentler [101]. We will assess the equivalence of the treatment groups on key baseline variables (demographics and psychological variables); variables on which the groups differ will be used as covariates in the final analyses. We will also examine attrition from the study as a dichotomous outcome and use logistic regression to determine whether treatment assignment predicts attrition.

#### Aims

##### Aim 1

- H1: Participants randomized to the EASE intervention will have greater reductions in anxiety, depression, and functional impairment at 3- and 6-month follow-ups compared to those randomized to the mindfulness/relaxation-based comparison intervention. Multiple approaches to modeling changes in primary outcomes will be used to evaluate our hypothesis that the EASE app will result in greater reductions in the primary outcomes relative to the comparison app and that reductions will be similar across racial/ethnic groups. We will first estimate between-group differences by calculating Cohen d effect sizes (with a 95% CI) for anxiety, depression, and functional impairment at each major follow-up assessment. We will then conduct a series of conditional LGMs to examine the impact of the treatment condition on the trajectories of primary outcomes from baseline to the end of the follow-up period. First, an LGM will be specified using the assessments of primary outcomes at major assessments: baseline, 3 months, and 6 months. A dummy code representing the treatment condition will be included in the model as a predictor of the intercept and slope factors to quantify the effect of the EASE program on the longitudinal course of outcomes relative to the comparison group. The statistical significance (and magnitude of the effect size) of the main effect of treatment condition will test H1 (the EASE app will show greater reductions in anxiety [OASIS] and depression [ODSIS] symptoms and improved functional impairment). Separate models will be specified for anxiety, depression, and functional impairment.
- H2: Intervention effects of EASE relative to mindfulness/relaxation-based comparison will be equivalent across racial/ethnic groups. We will next conduct similar effect size and LGM analyses within each of the 4 racial/ethnic groups in order to test H2 (the effectiveness of EASE will be similar across racial/ethnic groups). We will then use MSEMs to examine the effects of the EASE app on the longitudinal course of outcomes, as measured by the EMA daily diary data. MSEM analyses will also be conducted both across and within the racial/ethnic groups to evaluate how effects vary across groups.

##### Aim 2

- H3: Intervention effects on study outcomes listed in aim 1 will be mediated by reductions in AS and changes in COVID-19–related stress and fear. Similar modeling procedures will be used to determine the impact of the treatment condition on AS and COVID-19–related stress and fear and whether improvements in primary outcomes are mediated by reductions in AS and the secondary mechanisms. We will first conduct separate LGMs for AS and the secondary mechanisms using methods such as those described for the primary outcomes. After conducting univariate LGMs to explore changes in AS and secondary mechanisms as a function of treatment condition, we will conduct a series of parallel process LGMs to examine how changes in AS and secondary mechanisms relate to changes in the primary outcomes. The effects of changes in AS and secondary mechanisms on changes in the primary outcomes will be examined by specifying the slope factor for each potential mechanism as a predictor of the slope factor for each outcome. Separate parallel process LGMs will be conducted for each primary outcome, and a series of parallel process LGMs will be evaluated to determine the unique effect of each mechanism when modeled simultaneously. The indirect effects of treatment on primary outcomes via AS and other potential mechanisms will be evaluated by calculated bootstrapped CIs of the indirect effect using the MODEL indirect command in MPlus. These parallel process LGMs will not provide formal tests of mechanisms of change, as these models will be examining concurrent changes across the 6 months of assessment, but if the results from these models are promising, they will be followed by analyses that include the EMA measures of outcomes and mechanisms and will use models that permit more fine-grained examinations of temporal dependencies of change (ie, mediation models in MSEMs and latent difference score models).
- H4: Perceived discrimination (worse intervention outcomes), social support (better intervention outcomes), or the SES (lower SES associated with worse outcomes) will moderate intervention effects on study outcomes listed in aim 1. The moderating influence of discrimination, social support, and SES will be evaluated using conditional LGMs in which condition, moderator, and condition×moderator interaction terms will be specified as predictors of the slope factor for primary outcomes using the XWITH command in MPlus. Separate models will be specified for each hypothesized moderator, and conclusions will be based on the CIs of the interaction terms.

##### Exploratory Aims

The brief qualitative interviews will be transcribed following completion and read to ensure accurate transcription. The transcribed interviews will be coded using NVivo v.12 (QSR International). The study team (2-3 project staff) will create the initial codebook and then code 2 interviews together. The codebook will then be revised (if needed), and a third interview will be coded as a team. If there is high coding agreement between team members, the remainder of the interviews will be coded independently by the staff and then checked by author MC. The team will meet to discuss any disagreements in coding and to create a final coded version for thematic analysis. Themes will be determined within and across codes. Comparisons will also be made across racial/ethnic groups and between the intervention and comparison groups. An additional team member will then review the coding and analysis for confirming and disconfirming evidence for the themes [102]. Participant responses will be integrated with quantitative usage data to identify inconsistencies in the participant’s perceptions and actual engagement with the app [103,104]. The team will make recommendations that can be used to improve app engagement and modifications for specific racial/ethnic groups. MSEMs will then be used to further explore temporal patterns of COVID-19 outcomes and health behaviors using daily EMA data. Specifically, we will specify a series of models to examine interrelationships and concurrent and lagged effects among COVID-19 experiences (eg, treatments received, duration of symptoms), sequelae (eg, job loss, eviction, reduction in unemployment benefits), and health behaviors affected by COVID-19 (ie, physical activity, pain experience, sleep) among those who do and do not contract the virus.

#### Missing Data

We anticipate some participant attrition during the trial. Missing data will be handled in all analyses using direct ML techniques within MPlus under a missing-at-random (MAR) assumption [105]. Modern missing data techniques, such as direct ML, increase statistical power and provide more accurate estimates of model parameters and standard errors and are the recommended intent-to-treat approach for clinical trials.

#### Statistical Power

We calculated the sample size required to detect the treatment effect of EASE on the primary outcomes of anxiety, depression, and functional impairment at 3 and 6 months both across and within the racial/ethnic groups, the treatment effect of EASE on the longitudinal course of AS and secondary mechanisms, and the indirect effects of EASE on primary outcomes via the hypothesized mechanisms. Based on our pilot data and prior published research, we hypothesize moderate-to-large (d>.50) effects of EASE on primary outcomes and the hypothesized mechanisms and moderate-to-large associations (r>.30) between changes in mechanisms and primary outcomes. Power determinations based on these hypothesized effect sizes were conducted using GPower, RMASS [106], and simulation studies identifying the sample sizes needed to detect indirect effects [107]. Power analyses indicated that the targeted sample size of 200 participants for each of the 4 racial groups would yield greater than 80% power to detect effects for each study aim at α=.05, resulting in the overall target sample size of 800. Stratified block randomization was used to ensure equal allocation of adults across sex and racial/ethnic groups across treatment conditions.

## Results

The study was funded by the National Institute of Mental Health (NIMH) in May 2021. The smartphone app was finalized in December 2021, and data collection began on December 22, 2021. Study staff have since been engaged in activities associated with study enrollment and data collection, including ordering and monitoring of gift cards and supplies; screening, consenting, and enrolling participants; dispersing intervention incentives; follow-up tracking/calls; maintaining an up-to-date Institutional Review Board (IRB) protocol and associated documents; and monitoring incoming data to identify and resolve problems early (ie, both EMA and traditional questionnaire data). As of June 17, 2022, 217 participants have been recruited into the study.

## Discussion

### Principal Results

Mental health disparities across racial/ethnic groups are projected to become more pronounced as a result of the COVID-19 pandemic [9]. Emerging evidence points to the utility of a digitally delivered, mobile transdiagnostic intervention that engages AS for ameliorating anxiety and depression [49]. Integrating theoretical and empirical models with a practical approach to treatment delivery, the proposed study aims to address emerging and downstream mental health disparities among BHAI populations by testing whether our novel intervention (EASE) outperforms an active, credible comparison group. Data from this efficacy trial will determine whether EASE successfully improves symptoms of anxiety and depression and whether these improvements outperform an active comparison control app. If successful, EASE may be ready for implementation and dissemination into real-world behavioral health and social service settings consistent with the 4 objectives outlined in the NIH’s strategic plan [108]. Overall, this proposal has the potential to decrease anxiety and depression symptoms among all populations, including vulnerable populations determined to be most at risk of exacerbated, long-lasting negative health sequelae.

Notably, all study procedures are administered remotely. As such, this study may serve as a clinically important example for how to implement remote/virtual mental health research with BHAI individuals. Indeed, such flexible platforms for treatment delivery may have greater potential to reach and impact people of color, who constitute an underserved, hard-to-reach population. By providing a remote means of care, our study may be able to assess and assist a larger group of individuals who identify as BHAI and remove potential barriers to treatment access, such as money needed to commute into the office/clinic or the need to take time off from work to attend appointments.

### Limitations

One notable limitation of our study is the reliance on Android smartphones to complete assessments and access app features. Study-provided Android smartphones will be loaned to eligible participants upon request; however, those who are unwilling to use a study-provided smartphone or their own personal Android smartphone will be unable to participate in the study. Additionally, recruitment of older populations may be hindered due to the lack of appropriate knowledge and familiarity with mHealth apps [109]. This study will focus on enrolling individuals who identify as either Black/African American, Hispanic, American Indian/Alaska Native, or NHW; therefore, those who identify their racial/ethnic identity as an identity other than Black/African American, Hispanic, American Indian/Alaska Native, or NHW, such as identifying solely as Asian, Native Hawaiian, or Other Pacific Islander, will be excluded from the study. Given that these groups have faced elevated levels of xenophobia [110] and discrimination (eg, microaggressions, hate crimes) [111] due to the COVID-19 pandemic, it is important that these groups—and the unique issues they face—be addressed in the development of future interventions. The experimental group targets 1 transdiagnostic factor (AS); however, future research would benefit from evaluating other factors associated with COVID-19–related anxiety, distress, and disability. Further, although the app includes videos on the disproportionate effect the pandemic has had on BHAI individuals, the cultural tailoring of the app and its features (ie, tailored treatment messages, resources) would benefit from further development. Finally, we will conduct exploratory analyses of how the frequency/duration of app feature use may relate to outcomes. Given that all app features are available simultaneously, these exploratory analyses will be confounded by the potential effect of using other app features. Future work would benefit from conducting a dismantling study of EASE to understand the potential for each component to improve outcomes.

### Conclusion

Ultimately, the goal of this study is to target and reduce emerging and likely exacerbated mental health disparities among BHAI individuals. We aim to fill a void surrounding mental health treatment among all groups but particularly BHAI individuals by developing a digitally delivered, mobile, easy-to-use treatment for anxiety and depression. By addressing comorbid anxiety and depression symptoms through an intervention that targets an underlying transdiagnostic vulnerability factor (eg, AS), we hope that this intervention will help adults with anxiety or depression to reduce their symptoms.

## Abbreviations

6CIT: 6-Item Cognitive Impairment Test
AS: anxiety sensitivity
BHAI: Black, Hispanic, and American Indian
CBT: cognitive behavioral therapy
CRBS: Coronavirus Racial Bias Scale
EASE: Easing Anxiety Sensitivity for Everyone
EDS: Everyday Discrimination Scale
EMA: ecological momentary assessment
F-SozU K-6: 6-Item Perceived Social Support Questionnaire
LGM: latent growth model
mHealth: mobile health
ML: maximum likelihood
MSEM: multilevel structural equation model
NHW: non-Hispanic White
OASIS: Overall Anxiety Severity and Impairment Scale
ODSIS: Overall Depression Severity and Impairment Scale
RCT: randomized controlled trial
REALM-SF: Rapid Estimate of Adult Literacy in Medicine-Short Form
REDCap: Research Electronic Data Capture
SES: socioeconomic status
SSASI: Short-Scale Anxiety Sensitivity Index

## Data Availability

The data sets generated and analyzed during this study will be available from the corresponding author on reasonable request following the completion of the trial and publication of the main outcomes paper. Deidentified data also will be made available via the National Institute on Mental Health (NIMH) Data Archive (NDA).
